# Publication trends of research on sepsis and programmed cell death during 2002–2022: A 20-year bibliometric analysis

**DOI:** 10.3389/fcimb.2022.999569

**Published:** 2022-09-23

**Authors:** Jing-yan Li, Ren-qi Yao, Min-yue Xie, Qi-yuan Zhou, Peng-yue Zhao, Ying-ping Tian, Yong-ming Yao

**Affiliations:** ^1^ Department of Emergency, The Second Hospital of Hebei Medical University, Shijiazhuang, China; ^2^ Translational Medicine Research Center, Medical Innovation Research Division and Fourth Medical Center of the Chinese People's Liberation Army (PLA) General Hospital, Beijing, China; ^3^ Beijing Tongren Hospital, Capital Medical University, Beijing, China

**Keywords:** bibliometric analysis, publication, sepsis, programmed cell death, infection

## Abstract

**Background:**

Sepsis is considered an intractable dysfunction that results from the disordered host immune response to uncontrolled infection. Even though the precise mechanism of sepsis remains unclear, scientific advances have highlighted the key role of various programmed cell death processes in the pathophysiology of sepsis. The current study aims to explore the worldwide research trend on programmed cell death in the setting of sepsis and assesses the achievements of publications from various countries, institutions, journals, and authors globally.

**Material and methods:**

Associated publications during 2002–2022 with the topical subject of sepsis and programmed cell death were extracted from the Web of Science. VOSviewer was utilized to evaluate and map the published trend in the relevant fields.

**Results:**

All 2,037 relevant manuscripts with a total citation of 71,575 times were screened out by the end of 1 January 2022. China accounted for the largest number of publications (45.07%) and was accompanied by corporate citations (11,037) and H-index (48), which ranked second globally. The United States has been ranked first place with the highest citations (30,775) and H-index (88), despite a low publication number (29.95%), which was subsequent to China. The journal *Shock* accounted for the largest number of publications in this area. R. S. Hotchkiss, affiliated with Washington University, was considered to have published the most papers in the relevant fields (57) and achieved the highest citation frequencies (9,523). The primary keywords on the topic of programmed cell death in sepsis remarkably focused on “inflammation” “immunosuppression”, and “oxidative stress”, which were recognized as the core mechanisms of sepsis, eventually attributing to programmed cell death. The involved research on programmed cell death induced by immune dysregulation of sepsis was undoubtedly the hotspot in the pertinent areas.

**Conclusions:**

The United States has been academically outstanding in sepsis-related research. There appears to be an incompatible performance between publications and quantity with China. Frontier advances may be consulted in the journal *Shock*. The leading-edge research on the scope of programmed cell death in sepsis should preferably focus on immune dissonance-related studies in the future.

## Introduction

Sepsis is defined as a life-threatening syndrome that is attributed to a dysregulated host response to infection, eventually leading to high mortality and morbidity in critical illnesses (Mayr et al., 2014; Patil et al., 2017). According to the constitutive definition for Sepsis 3.0, which revised the pathogenic septic shock as lethal multiple-organ dysfunction, the keynote on sepsis-related mechanisms mainly focuses on the disturbing immunoreaction to infection (Singer et al., 2016; Rhodes et al., 2017). In spite of increasingly prominent achievements in early identification, management, and intervening measures, sepsis remains an intractable challenge in emergent and severe cases (Czura, 2011). Unoptimistically, several clinical efforts to strengthen anti-inflammatory measures have failed to improve survival over the past decade, and there is no specific treatment for sepsis at present (Zeni et al., 1997).

It is acceptable that the interplay between immune dysregulation and host immune response might be involved in the pathogenesis and progression of sepsis, which has been the hotspot in near future. This evidence has been objectively summarized by Yao et al. in 2020, which showed a publication trend of research on sepsis and host immune response (Yao et al., 2020). Except for the abovementioned viewpoint, emerging evidence implies that sepsis initiates a complex network including the innate immune system and neuroendocrine system owing to the excessive inflammatory response (Hotchkiss et al., 2013a). In general, it is inevitable for septic patients to suffer from a long-term immunosuppressive state, which is closely related to prognosis and mortality. Intrinsically, sepsis-induced immune dysregulation results from the dysfunction of immune cells, such as T lymphocytes, B lymphocytes, and dendritic cells (DCs), which have been considered one of the primary causes for the exasperate outcomes with septic patients. Recently, an improved viewpoint underlying systemic inflammation as well as oxidative stress is highlighted to be involved in the pathogenesis of sepsis, which might be an initiator to trigger programmed cell death (Bajčetić et al., 2014; Meo et al., 2016). Indeed, an increasing number of academic teamwork have paid close attention to the effect of sepsis-induced immune dysfunction on programmed cell death, and corresponding trials for medical treatment have proceeded (Hotchkiss et al., 2019). There is no doubt that apoptosis, considered the key mechanism of immune cells with programmed death in a septic setting, has been the research emphasis in the past few years, which is mediated by the caspase-1 and programmed cell death ligand 1 (PD-L1) pathway (Chang et al., 2014). As shown in some clinical trials, excessive apoptosis of T lymphocytes, B lymphocytes, and DCs was confirmed as the central reason for poor prognosis of septic patients, thus attributing it to immune deficiency (Hotchkiss et al., 2002; Felmet et al., 2005). Likewise, inhibitory immune receptors are demonstrated to upregulate in the pathological process of sepsis, which leads to the disorder of the immune system, thereby hindering the defensive system from resisting foreign invasion (Zhang et al., 2011; Patil et al., 2016). Notably, many other types of programmed cell death (except for apoptosis) such as pyroptosis, necroptosis, ferroptosis, parthanatos, entotic cell death, and NETotic cell death, are confirmed to be involved in the pathological process of sepsis (Galluzzi et al., 2018). It is known that pyroptosis is attributed to rapid plasma-membrane fracture due to the non-selective gasdermin-D pore and inhibition of inflammasome, which ultimately results in neutrophil depletion in the occurrence of sepsis (Bergsbaken et al., 2009). Under the challenge of continuous release of the proinflammatory cytokine, destructive membrane permeabilization together with excessive lipid peroxidation might drive necroptosis to ferroptosis, which is thought to be involved in sepsis-associated renal failure (Friedmann et al., 2014). Additionally, a respiratory burst of immune cells is generally accompanied by NETosis, depending on the NADPH oxidase and reactive oxygen species (ROS) when sepsis occurs (Fuchs et al., 2007). Therefore, different types of cell death underlying the exact role and its regulatory mechanisms in sepsis remain currently unclear, and more efforts need to be made to explore the potential targets.

A bibliometric study has been reported as a comprehensive analysis to depict the detailed trend of topic research in the relevant field over a certain timeline (Dabi et al., 2016). It takes advantage of literature metrology to analyze the quantity and quality of publications depending on the literature system (Avcu et al., 2015). Since bibliometric analysis can take advantage of detailed document literature to evaluate the whole contribution derived from different countries, institutions, journals, and authors, it is beneficial to present and predict the development as well as a future trend (Narotsky et al., 2012). Of note, bibliometric studies are becoming more popular and may play an important role in the formulation of expert consensus, therapeutic prospects, and implementation of guidelines (Seriwala et al., 2015).

With the widespread acceptance of bibliometric subjects for literature analysis in recent years, increasing related topics on immunotherapy and early identification have been summarized. However, bibliometric analysis on sepsis and programmed cell death remains rarely reported to date. Herein, the present study aimed to provide an optimal analysis involving sepsis and programmed research by virtue of the Web of Science (WOS) in its current status. The relevant research trends as well as an underlying hotspot in certain fields would also be revealed.

## Materials and methods

### Data sources and search strategies

**Figure 1:**
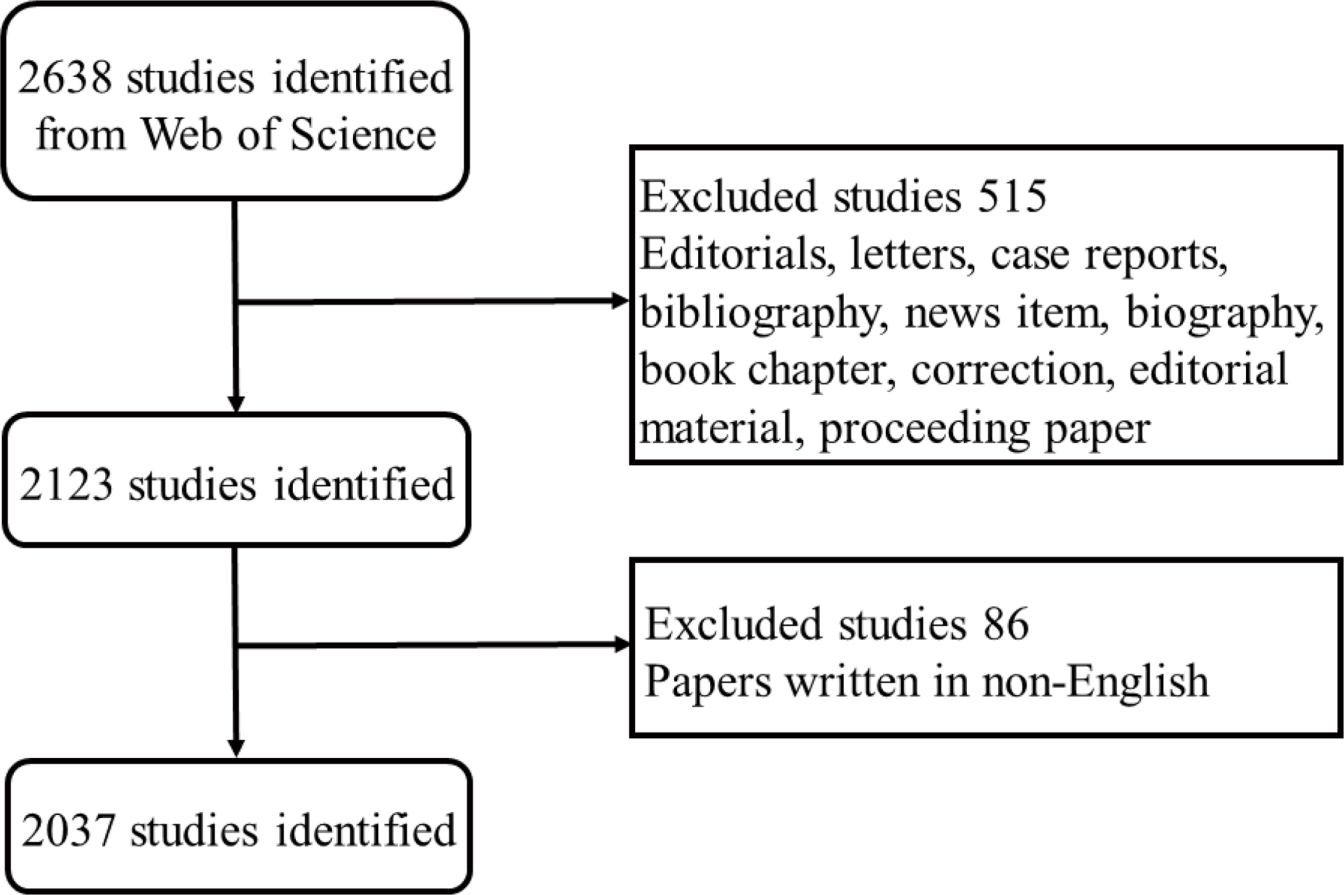
Flow diagram of the bibliographic retrieval process. The procedures of screening and enrollment are presented in detail.

Bibliometric analysis was conducted by applying the Science Citation Index-Expanded (SCI-E) of Web of Science Core Collection (WoSCC, Clarivate Analytics, 1988 to present, http://lib.tmu.edu.cn/), which is currently containing more than 12,000 of the highest-impact international journals and is confirmed as the most prominent scientometric databases of peer-reviewed scientific publications on related topics (Wu et al., 2021). All appropriate publications with paper types limited to research articles and reviews during the period from 2002 to 2022 were searched online in WOS. The corresponding retrievals were accomplished at a regular time on a fixed day to prevent statistical bias resulting from database updates. Among these, as sepsis was the primary research subject of the current study, in order to achieve a more precise result, terms related to “sepsis” was searched based on the title (TI). The search strategies were listed as follows: # 1, title: (sepsis* OR sepsis shock* OR endotoxemia* OR SIRS OR systematic inflammatory response syndrome*); # 2, topic: (programmed cell death OR regulated cell death OR Ferroptosis OR Apoptosis OR Pyroptosis OR NETosis OR PANoptosis OR Oxeiptosis OR Alkaliptosis OR Autosis OR Parthanatos OR Entosis OR Necroptosis OR Netotic cell death OR lysosomal cell death OR Autophagy-dependent cell death); # 3, “# 1” AND “# 2”. Wildcard “*” refers to any group of characters or no character; for example, “sepsis” would also return “septic” (Wu et al., 2021; Wu et al., 2021). Given that, other document types of literature were excluded accordingly except for articles and reviews, and the screening approach of enrollment and exclusion is presented in **Figure 1**.

### Data collection

Detailed data including titles, keywords of published articles, publications of a fixed number of years, countries and regions, authors, institutions, publishing journals, citations, and H-index were independently extracted from WOS. All the above information was imported into Microsoft Excel 2016 (Redmond, WA, USA), GraphPad Prism 8.0 (GraphPad Prism Software Inc., San Diego, CA, USA), and VOSviewer software (Leiden University, Leiden, Netherlands) for subsequent qualitative and quantitative analyses.

### Bibliometric analysis

It is widely accepted that the WOS database is appropriated for characteristic description with all incorporated publications. Relative research interest (RRI) is reflected by a ratio between the published number in a certain field and publications across all fields per year (Gao et al., 2017). Impact factor (IF) is obtained from the latest version of Journal Citation Reports (JCR). H-index is defined as the academic influence of a scholar or a country, which indicates a scholar/country has published H literature, each of which has been cited in other publications at least H times (Hirsch, 2005). VOSviewer is thought to be the appropriate approach for correlation analysis between the highly cited references with source publications as well as their institutions. Furthermore, it can visualize the network of keywords in the manner of color-coded clusters, which are classified on the basis of co-occurrence analysis (Synnestvedt et al., 2005). In addition, the average appearing years (AAY) is applied to indicate the relative originality of a keyword.

## Results

### Contribution of countries to global publications

**Figure 2:**
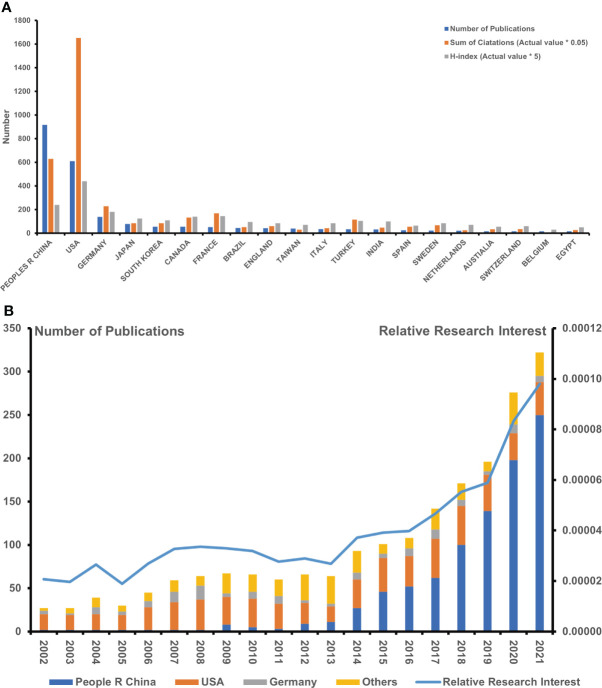
Achievement of various countries/regions in the research field regarding sepsis and programmed cell death. **(A)** The published number, summary citation frequency (×0.05), and H-index (×5) in the top 20 countries. **(B)** The published number globally and in the top 3 countries each year, and the timeline of relative research interest of sepsis and programmed cell death (*means "×").

**Figure 3:**
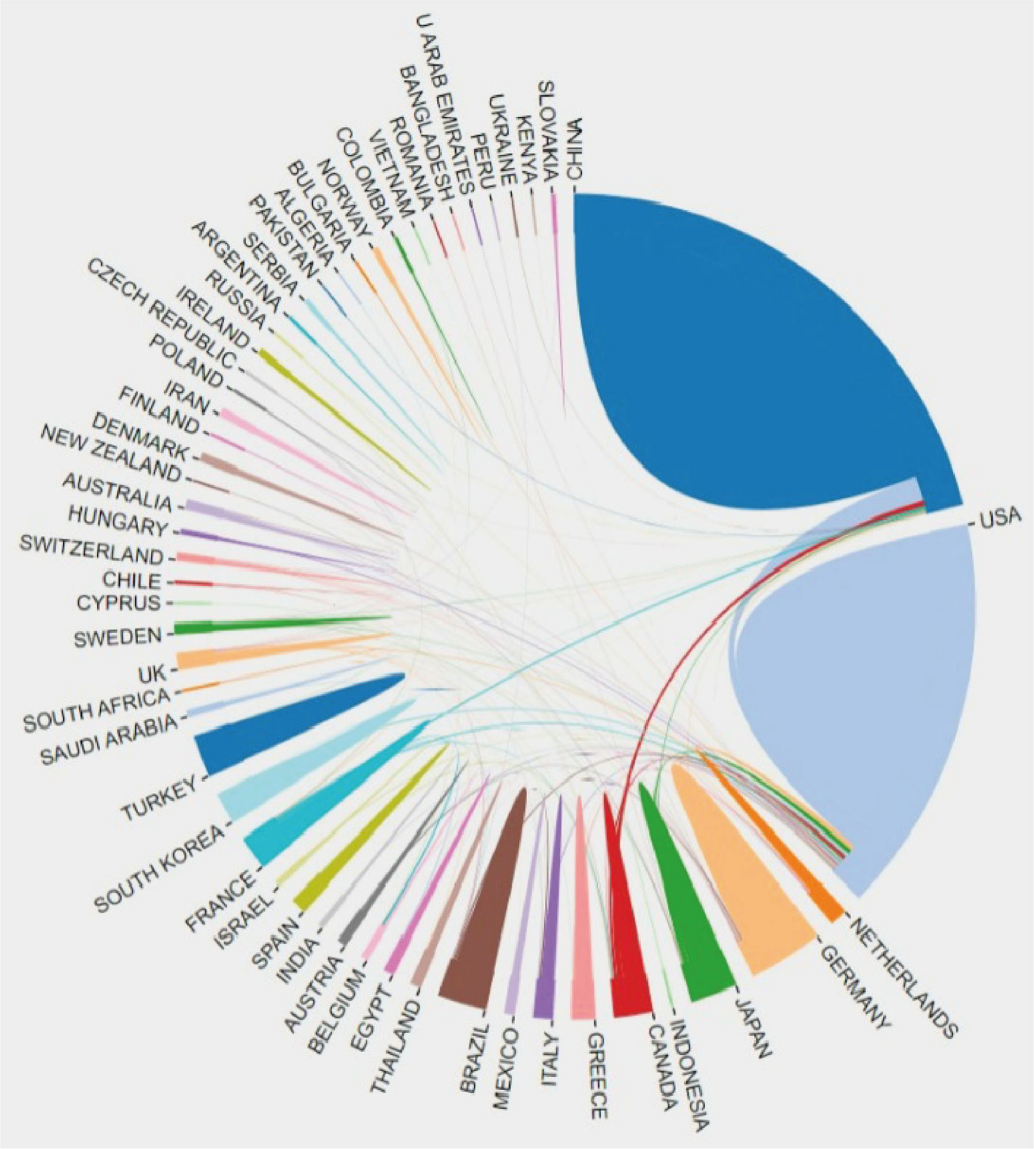
The cooperation of countries/regions in research scope on sepsis and programmed cell death from 2002 to 2022.

A total of 2,037 publications from 2002 to 2022 conformed with the inclusion criteria. China ranked first with respect to the number of publications (NP) (918, 45.07%), followed by the United States (610, 29.95%) and Germany (139, 6.82%), which was followed by Japan. In addition, South Korea, similar to Canada and France, has contributed to the same number of publications, falling behind Germany. The number of papers within 2021 (322, 15.8%) was indicated to be the most when comparing annual publications (**Figure 2A**). In consideration of publications from the holistic research field, the global concerns in this specific area, as presented by RRI, hovered around 0.005% before 2017, whereas they trended toward 0.008% during 2020 (**Figure 2B**). It showed that Chinese scholars firstly participated in this field in 2009, while relevant manuscripts in such topics had increased over the subsequent years. Meanwhile, China (46, 45.5%) seemed to be superior to the United States (39, 38.6%) in terms of NP after 2015. To gain insight into the cooperative relationship between worldwide countries, we applied VOSviewer online platform to visualize the connected network. Notably, the United States had made the most interpersonal cooperation with different countries globally, as the United States is situated in the central position of the network, as shown in **Figure 3**.

### Citations and H-index analysis

In line with the citation report retrieved from the database, the total citation frequency of all incorporated records reached 71,575 times since 2002 (67,427 times without self-citations), which was equivalent to 33.1 times per paper. Although China was the leading country with the most NP, its total citation (TC) (17.56%) together with an H-index of 48 fell behind that of the United States (46.17%) with an H-index of 88, which ranked the first among all countries. Germany ranked third, with citations of 6.39% and an H-index of 36 that followed behind China. Germany was followed by France, which possessed a high citation of 168.85. Notably, Canada and Turkey ranked fifth and sixth, respectively, with high citations of 133.3 and 116.2 (**Figure 2A**). Of note, the publication garnering the highest citation is a commentary piece in *Lancet Infectious Diseases.* Among the top 10 highly cited papers, five were primary articles, and the remaining five publications were reviews.

### Growth trends of publications

**Figure 4:**
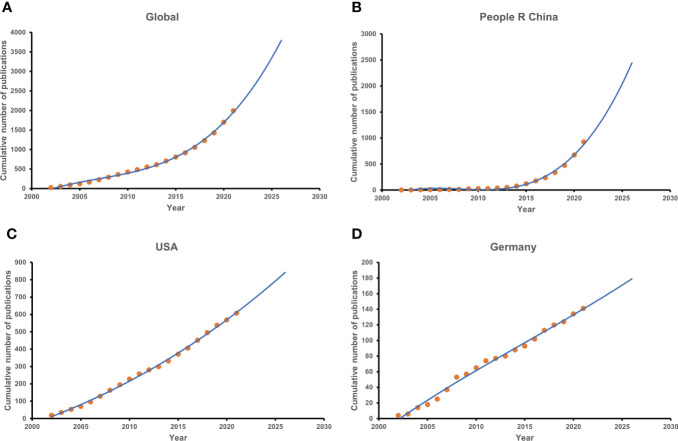
The model fitting curves of growth trends with regard to sepsis and programmed cell death. **(A)** Global. **(B)** The People’s Republic of China. **(C)** The United States. **(D)** Germany.

Up to 2022, the model fitting curves of publication growth predicted that the cumulative publications worldwide would be 2,476 papers, in which China contributed approximately 924 papers, whereas roughly 607 articles came from the United States (**Figure 3**). It suggested that the global growth of publications has a slowly rising trend on the curve (**Figure 4A**), which coincided with dominating countries such as the United States and Germany (**Figures 4C, D**). On the contrary, the published papers in China were demonstrated to rise more rapidly in comparison to those in other countries (**Figure 4B**).

### Journals publishing studies on sepsis and programmed cell death

**Figure 5:**
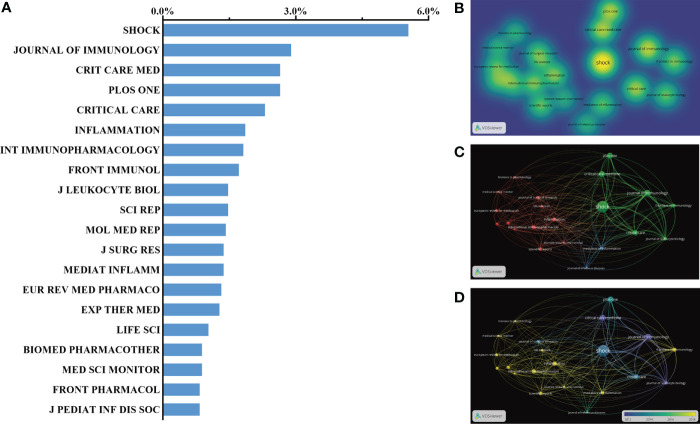
Distribution of published journals with respect to sepsis and programmed cell death. **(A, B)** Distribution of top 20 journals publishing research on sepsis and programmed cell death. **(C)** The network and relation between the top 20 journals. **(D)** Distribution of published journals is shown according to the average time. The blue color represents the early years, and the yellow color represents recent years.

Based on the search report with respect to journal sources, there was approximately one-third of articles published in the top 20 journals (726, 35.64%). According to the statistics, the leading journal with the highest NP was *Shock*, with 113 records (IF = 6.1, 2021), followed by the *Journal of Immunology* (IF = 5.442, 2021) with 59 records. Furthermore, *Critical Care Medicine* (IF = 12.7, 2021) had published as many papers as *PLOS One* (IF = 5.3, 2021) with 54 publications. A total of 47 papers had been published on *Inflammation* (IF = 6.0, 2021), which ranked fifth in the top 20 journals. Other journals, such as *Frontiers in Immunology* and *Molecular Medicine Report*, which were also representative of the academic impact in the relevant field, had contributed excellent-quality subjects, ranking in the top 10. Here, the top 20 journals that contributed the largest number of articles are listed in **Figure 5A**. In addition, the density map of the published journal is shown in **Figure 5B**, in which light yellow indicated a large number of published papers. A visible network presented in **Figure 5C** was constituted when similar subject words co-occurred in the same journal. The map of the time shaft in **Figure 5D** is representative of a journal-associated cluster with different colors according to the published year.

### Institutions publishing studies on sepsis and programmed cell death

**Figure 6:**
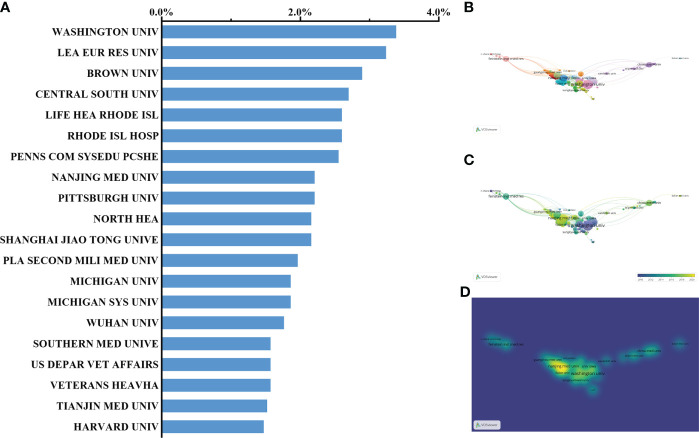
Distribution of institution-published articles regarding sepsis and programmed cell death. **(A, B)** Distribution of the top 20 institutions publishing research on sepsis and programmed cell death. **(C)** The network of collaboration between the top 20 institutions. **(D)** Distribution of published institutions is presented in accordance with the average time. The blue color represents the early years, and the yellow color represents the recent year.

Washington University in the United States with the highest NP (57, 2.79%) has the leading step among institutions worldwide. Of note, the institutions affiliated with the United States reached up to 12 within the list of top 20 organizations, followed by seven Chinese institutions and only one French institution. In fact, one of the Chinese institutions named *Central South University* has a total of 55 papers and ranked third, accounting for 2.7% of the total number of publications since 2009 (**Figure 6A**). Nevertheless, the number of publications from both *Nanjing Medical University* and *Fudan University* of China has gradually increased in recent years (**Figures 6B–D**).

### Authors publishing research on sepsis and programmed cell death

Approximately 218 papers with a proportion of 10.7% in the total publications were published by the top 5 authors, 57 of which were published by R. S. Hotchkiss and came from Washington University. A. Ayala from Rhode Island Hospital and Brown University published 47 papers in this field, followed by C. S. Chung with 41 publications accounting for 2.0%. C. M. Coopersmith and P. Wang had published 39 papers and 34 papers in total and ranked fourth and fifth, respectively. As listed in **Table 1**, all the top 5 authors were from the United States, which proved that the United States has a dominating role in the sepsis-associated research area. It is noteworthy that R. S. Hotchkiss is the most influential scholar in this field, as supported by the highest TC among all authors (9,523 times in total). Moreover, the publication of a review published on *Lancet Infectious Disease*s by Didier Payen gained the highest TC, while the primary article by R. S. Hotchkiss in the journal *Immunity* acquired the highest TC among the related research scope of sepsis and programmed cell death (**Table 2**).

**Table 1:** Top 5 authors with most publications in research scope of sepsis and programmed cell death.

Authors	Country	Affiliations	No. of publications	No. of citations
R. S. Hotchkiss	USA	Washington University	57	9,523
A. Ayala	USA	Brown University	47	2,788
C. S. Chung	USA	Brown University	41	2,448
C. M. Coopersmith	USA	Emory University	39	1,860
P. Wang	USA	Northwell Health	34	882

**Table 2:** Top 10 highly cited papers related to sepsis and immune response.

Titles	Corresponding authors	Journal	Publication type	Publication year	Total citations
Immunosuppression in sepsis: a novel understanding of the disorder and a new therapeutic approach	Didier Payen	*Lancet Infectious Diseases*	Review	2013	834
Sepsis and septic shock	J. L. Vincent	*Nature Reviews Disease Primers*	Review	2016	511
The pathogenesis of sepsis	D. G. Remick	*Annual Review of Pathology: Mechanisms of Disease*	Review	2011	370
Mechanisms of cardiac and renal dysfunction in patients dying of sepsis	R. S. Hotchkiss	*American Journal of Respiratory and Critical Care Medicine*	Article	2013	284
The immune system’s role in sepsis progression, resolution, and long-term outcome	P. A. Ward	*Immunological Reviews*	Review	2016	267
Advances in the understanding and treatment of sepsis-induced immunosuppression	G. Monneret	*Nature Reviews Nephrology*	Review	2018	217
The endotoxin delivery protein HMGB1 mediates caspase-11-dependent lethality in sepsis	B. Lu	*Immunity*	Article	2018	183
Lipid peroxidation drives gasdermin D-mediated pyroptosis in lethal polymicrobial sepsis	D. L. Tang	*Cell Host and Microbe*	Article	2018	154
Chemical disruption of the pyroptotic pore-forming protein gasdermin D inhibits inflammatory cell death and sepsis	D. W. Abbott	*Science Immunology*	Article	2018	138
Immune checkpoint inhibition in sepsis: a phase 1b randomized, placebo-controlled, single ascending dose study of anti-programmed cell death-ligand 1 antibody (BMS-936559)	D. M. Grasela	*Critical Care Medicine*	Article	2019	80

### Analysis of keywords in publications on sepsis and immune response

**Figure 7:**
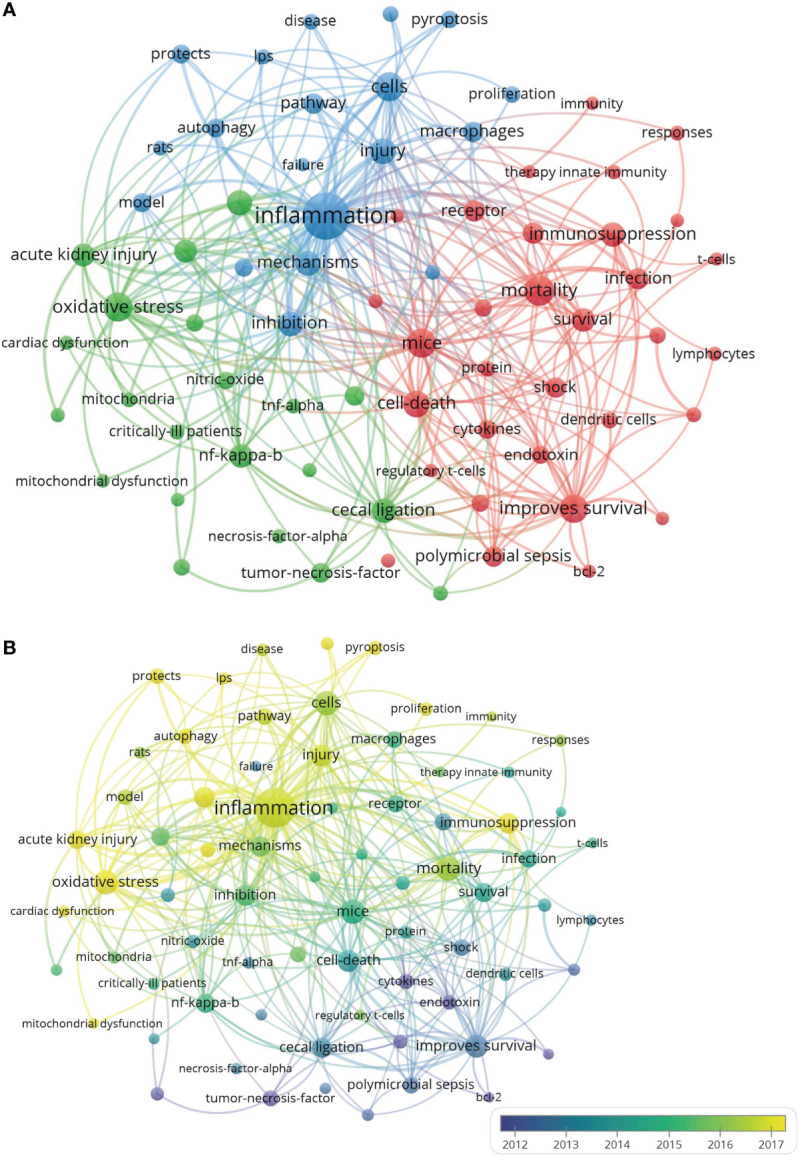
The analysis of keywords in publications on sepsis and programmed cell death. **(A)** Mapping the keywords within the field of sepsis and programmed cell death. All the keywords were classified into three clusters in line with different colors: oxidative stress-related research (left, in green), immunosuppression-related research (right, in red), and inflammation-related research (top, in blue). The larger cycle represents the keywords that presented with a higher frequency. **(B)** Distribution of keywords is shown in accordance with the appearance for the average time. The blue color represents words during the early stage, while the yellow color represents the words that appeared in recent years.

As shown in **Figure 7A**, the whole keywords extracted from the title and abstract of 2,037 appropriate publications were analyzed and clustered by VOSviewer. By visualizing keywords with high co-occurrences defined as items that existed more than 32 times, 100 keywords were classified into three mechanism-associated subclusters: “immunosuppression” (red), “oxidative stress” (green), and “inflammation” (blue). Within the cluster of “immunosuppression-related research”, relevant keywords included improved mortality (203 times), mice (197 times), improved survival (179 times), cell death (160 times), and immunosuppression (139 times). In the cluster of “oxidative stress-related research”, the main keywords were acute lung injury (143 times), cecal ligation (138 times), lipopolysaccharide (121 times), endotoxemia (69 times), and nitric oxide (67 times). As with the cluster of “inflammation-related research”, the major words were presented as follows: cells (195 times), injury (159 times), inhibition (150 times), mechanisms (142 times), and pathway (98 times). Detailed information with regard to the co-occurrences of keywords is listed in **Supplementary Table S1**.

As illustrated in **Figure 7B** and **Supplementary Figure 1**, all the incorporated keywords were color-coded according to the average number of times when the word appeared. The words presented in the early phase of research might be tinged in blue, while the recent ones would be marked with yellow. During the early stage of the study with regard to sepsis and programmed cell death, “endotoxin” with AAY of 2,013.536 in cluster 1 represented the mainstream in the relevant field. The latest research mainly focused on “lymphocyte apoptosis” (cluster 1, AAY was 2,011.767), which might be the new target for a topic on sepsis and programmed cell death. In the first cluster (immunosuppression-related research), the most updated word was “T cells” (cluster 1, AAY was 2,014.886), which appeared approximately 44 times. With the second cluster (oxidative stress-related research), the word “mitochondrial function” (cluster 2, AAY was 2,016.954) had been confirmed as the recently present word that emerged 43 times. As with the third cluster (inflammation-related research), “pyroptosis” (cluster 3, AAY was 2,019.747) was noted as a novel topic, which had emerged 71 times.

## Discussion

### Research trends in sepsis and programmed cell death

The present research indicates that China has made the greatest contributions with respect to the number of publications on the topic of sepsis-associated programmed cell death. However, the United States has been confirmed to be the leading country in citation frequency as well as H-index value in relevant fields, which might be in accordance with the fact that the United States has been concerned more early with the mechanism of programmed cell death with the septic model than had other countries. Meanwhile, the United States appears to be the leading country in academic research and clinical trials, as evidenced by its advanced equipment, exquisite technology, professional team, and abundant funds.

Notably, China has been superior to other countries, with the largest number of papers, but it fell behind in terms of citation frequency. The reason that a distinct discrepancy between quantity and quality with publications from China occurred might be listed as follows. Firstly, scholars from China have focused their attention on the area of programmed cell death in sepsis only starting in 2009; as a result, they have a low number of published articles before 2015. Thus, it is a great challenge for China to publish more high-quality articles. Moreover, there is a universal phenomenon that the omission diagnostic rate remains high in China than in other developed countries, resulting from non-standard diagnosis as well as inconsistent systematic scores, which eventually cause the deficiency of clinical documents.

As indicated in the time curve, the globally relevant field presented a rapid growth period in published numbers since 2014. Of note, the number of Chinese publications continued to progress quickly until it surpassed that of the United States in 2015 when the other countries had reached a stable stage. Moreover, the published number during the recent 5 years markedly exceeded the total publications, of which Chinese papers accounted for a large proportion assuredly. Among the top 20 institutions, 12 authoritative institutions affiliated with the United States contributed to the present field, indicating their advantage over the sepsis-associated research area. A French institution named the League of European Research Universities (LERU) ranked second in the top 20 institutions. Notably, there are seven Chinese institutions located in the south of China, suggesting the imbalance of areal distribution in the research field. Thus, more professional teams from other developed countries as well as international collaboration seem to be required in future studies. Taken together, the United States is equipped with the most elite institutions globally, which is consistent with their dominant status in research scope regarding sepsis and programmed cell death.

It was noteworthy that the number of publications from the journal *Shock*, which is generally accepted as the most representative journal, had reached 113. Other major journals, such as the *Journal of Immunology*, *Critical Care Medicine*, and *Critical Care*, were also involved in the publication of programmed cell death resulting from sepsis. Therefore, it is our belief that more leading-edge studies concerning the related topic might be acquired from the abovementioned excellent journals.

As with the relevant authors, R. S. Hotchkiss from the United States published the most papers on sepsis and programmed cell death with the highest citations in the list, followed by both A. Ayala and C. S. Chung, who also came from the United States. R. S. Hotchkiss, who is considered the pioneer in the pathogenesis of sepsis-induced immune dysregulation, has made great contributions to research on sepsis and apoptosis of immune cells, evidenced by the unexpected citation frequency (Hotchkiss et al., 2013a; Hotchkiss et al., 2019). The investigation from A. Ayala primarily focused on the dysfunction of immune cells in the different periods when sepsis progressed (Wisnoski et al., 2007; Huang et al., 2009; Venet et al., 2009). C. S. Chung mainly inquired into the novel role of programmed cell death receptors in sepsis-mediated organ dysfunction and made more effort to explore the regulated pathway in immune cell death caused by septic challenge (Wang et al., 2016; Wu et al., 2017). Additionally, both C. S. Chung and G. Monneret, who were regarded as co-authors in various manuscripts, revealed the important collaborations between these institutions, which indicated that teamwork is an efficient strategy for academic exchange (Rossi et al., 2019). It is firmly believed that the outstanding scholars might make more contributions to the hotspot of this field, which plays a key role in further exploration of targeting for septic complications.

### Research focuses on sepsis and immune response

There is no doubt that publications with the highest citation times are closely related to great academic influence in the professional field. The detailed information from the top 10 cited publications, including titles, corresponding authors, journals, and citations, are listed in **Table 2**. The manuscript titled “Immunosuppression in sepsis: a novel understanding of the disorder and a new therapeutic approach” was published in the journal *Lancet Infectious Diseases*, which has the highest citation frequency of 834 times. This work team with the corresponding author named Didier Payen found that taking advantage of interleukin (IL)-7, an immune-mediated modulator in anti-programmed cell death, might be the future in the treatment of sepsis, evidenced by the suppression of cytokine as well as the exhaustion of T cell (Hotchkiss et al., 2013b). In 2016, for the subsequent research, R. S. Hotchkiss reviewed the current epidemiology of septic shock and the practice for the recognition and utilization of biological response modifiers in septic patients, entitled “Sepsis and septic shock”, with the corresponding author J. L. Vincent (Hotchkiss et al., 2016). In fact, as many as four papers of the top 10 publications were attributed to R. S. Hotchkiss and his colleagues, which were the reasons that he had gained the highest citations in this specific scope. It is noteworthy that the first three publications in the top 10 highly cited papers related to sepsis and immune response are reviews and even commentaries, while the others are primary articles, which showed a stronger generality and higher citation in reviews.

As for the latest hotspot, “pyroptosis” from the “inflammation-related research” in cluster 3 showed a total of 71 times, indicating that a novel type of programmed cell death was involved in the sepsis-associated field. Within the five most newly occurred keywords, there were three words including “protects”, “autophagy”, and “pyroptosis” from “inflammation-related research”, while the other two words (“cardiac dysfunction” and “acute kidney injury”) originated from “oxidative-related research”. Although the immune dysregulation-associated cluster seemed to have drawn less attention when compared with the others, this cluster mainly focused on “mortality” and “survival”, which suggested that deep insight into the underlying mechanism of immune dysregulation might improve the prognosis of septic patients and be widely accepted in the future.

However, it is worth noting that programmed cell death involves various forms, such as ferroptosis, pyroptosis, apoptosis, and especially necroptosis. Up to now, both ferroptosis and pyroptosis have been reportedly paid much more close attention to by most researchers globally. According to the current analysis report on publication trend, ferroptosis and pyroptosis in the septic setting were confirmed as the hottest topics especially in China and the United States, as evidenced by manuscripts on pyroptosis in sepsis accounting for 12.2%, which were followed by the topic with regard to ferroptosis under exposure to sepsis with a proportion of 8.1%. Among these publications, research orientation mainly depended on immunology, cell biology, and biochemistry molecular biology since 2017 worldwide. Moreover, it is supposed that pyroptosis presented a more close contact with sepsis than ferroptosis, resulting from the release of the inflammasome in the process of pyroptosis when sepsis occurred. This might be the major cause for publications on pyroptosis in sepsis that achieved the highest citation by the team of D. L. Tang. Nevertheless, ferroptosis identified as the newly discovered cell death has been intensively a concern by most scholars, contributing to its peroxidative response in sepsis, which will further promote an upsurge in research on ferroptosis and sepsis.

In fact, the essential mechanism of the pathological process in sepsis largely appears to be associated with immune dysregulation, attributed to oxidative stress and excessive inflammation, and it is in accordance with the three clusters shown in VOSviewer, eventually resulting in programmed cell death. It is now widely accepted that the initial hyperinflammatory phase followed by the chronic immunosuppressive stage might be involved in the pathogenesis and pathophysiology of sepsis (Nedeva et al., 2019). Thus, the severe degree of immune cell death is proven to be closely related to the mortality of sepsis, even though the immediate cause remains unknown. For example, a study on a septic mouse model revealed that splenectomy together with lymph organ elimination could effectively protect mice against secondary sepsis, which confirmed splenocyte death to be potentially pathogenic in sepsis (Huston et al., 2008; Drechsler et al., 2018). Other than apoptosis highlighted in the last two decades, other types of programmed cell death have been recently emerging in the field of sepsis, such as pyroptosis, ferroptosis, parthanatos, entotic cell death, and NETotic cell death. Neutrophilic pyroptosis resulting from plasmalemma fracture caused by non-selective gasdermin-D pore has been documented as the primary impact influence on sepsis (Singla and Machado, 2018; Liu and Sun, 2019). Along with the process of sepsis, the occurrence of ferroptosis in immune cells attributed to excessive lipid peroxidation may lead to increased membrane permeability in necroptosis and release of particular damage-associated molecular patterns (DAMPs), which can convert to other types of cell death if the stimulation remains persistent (Jorgensen et al., 2017). Both respiratory burst and NETosis in immune cells involving the accumulation of ROS and NADPH oxidase exert an important impact on sepsis (Brinkmann et al., 2004). As for oxidative stress in programmed cell death during sepsis, the critical pathology depends on excessive ROS induced by lipid peroxidation, which destroys biomembranes, propagates oxidative chain reactions, and results in cell death in the following process. In fact, the production of peroxidation interacts with transport receptors to activate the apoptosis-mediated signaling pathway (Chaudhary et al., 2010; Elkin et al., 2018). ROS may also stimulate the peroxidative response of cardiolipin, which is a phosphatidylinositol located in mitochondria, in turn leading to intrinsic apoptosis (Zhong et al., 2017). Iron-mediated ROS is essential to activate ferroptosis when sepsis occurs, and ROS accumulation can be primarily produced in the mitochondrial energy metabolism as well as Fenton’s reaction during the process of iron transport (Doll and Conrad, 2017). Accordingly, iron chelators with the main mechanism of regulating intracellular iron metabolism effectively defend plasmalemma against lipid peroxidation by decreasing iron utilization in the iron pool (Kruszewski, 2003). Thus, it seems that both Fenton’s chemical reaction and iron-dependent lipoxygenases may improve peroxidative reaction, which can further exacerbate the free-radical injury, thereby resulting in ferroptosis-associated cell death.

With respect to the effect of overwhelming systemic inflammation on programmed cell death in the setting of sepsis, it mainly depends on the imbalance of inflammation and immune dysregulation, as evidenced by the excessive inflammatory response at the early stage whereas a sign of immunological paralysis with high risk for the secondary infections in the later period. The infectious cells release a large amount of dangerous mediators, which stimulate the production of proinflammatory cytokines, finally leading to apoptosis of immune cells (Bone, 1996). Unless the apoptotic cells are instantly identified and captured by macrophagocyte, they are likely to undergo secondary necrosis, releasing enterocytes and inducing an inflammatory response (Rock and Kono, 2008). Interestingly, a bacterial infection is capable of stimulating immune cells especially macrophages, dendritic cells, and B lymphocytes to activate the apoptotic pathway, which accelerates the anti-inflammatory response (Kobayashi et al., 2003). Additionally, pyroptosis has been proposed as a particular programmed cell death with a large amount of inflammasome induction, which promotes dimerization of inflammatory response-mediated caspases, resulting in the formation of active proteins, the processing of IL-1β, and the initiation of the signaling pathway (Fink and Cookson, 2007). Inflammasomes, noted to be a category of cytoplasmic multi-protein complexes, selectively perceive DAMPs (Broz and Dixit, 2016). In particular, the nucleotide-binding oligomerization domain (NOD)-like receptor protein 3 (NLRP3) inflammasome is noticed as the most comprehensively characterized inflammasome, and it augments the activation of caspase-1 through recruitment of the adaptor apoptosis speck-like protein (Elliott and Sutterwala, 2015). Strikingly, excessive activation of inflammasome induces injury and dysfunction of immune cells especially of multiple organs, contributing to the development of sepsis and septic shock (Man et al., 2017). Collectively, deep insight into cellular fate especially programmed cell death from the perspective of immune dysregulation, oxidative stress, and extensive inflammation is indeed essential to explore the novel mechanism underlying sepsis and targeting for functional homeostasis of modulatory pathways.

### Strengths and limitations

The current analysis is relatively comprehensive and accurate as evidenced by the extracted data from the WOS database. Nevertheless, certain limitations remain, as follows. Indeed, we have taken notice of the global COVID-19 pandemic from 2020 to 2022 for reason that some severe COVID-19 patients may proceed with sepsis. However, fundamental studies on COVID-19-induced sepsis remain deficient since related studies mainly focus on clinical trials. Although the reliability and authenticity of the conclusion might not be impacted, we are continuously concerned about the fundamental experimental studies of COVID-19-induced sepsis to further verify the research. Moreover, it is assumed that sepsis-related topics include multiple emphases, and various mechanisms are involved in the pathogenesis and progression of sepsis, such as excessive inflammatory response, internal environment disturbance, microcirculation dysfunction, and immune dysregulation. Even so, it may be unable to eliminate statistical bias as the reviewer described, attributing to the possibility that part of the publications is focused on microenvironment or metabolic balance rather than an immune-related perspective. In line with the inclusion criteria, non-English relevant publications are inevitable to be excluded from the analysis, which might result in research bias. Meanwhile, periodic renewal of the database may cause study flaws, which should be improved by conducting the search works within 1 day. Additionally, the types of research that met the inclusion criteria were limited to articles and reviews. Thus, letters, conference papers, and books, which may have an academic impact, were excluded. Moreover, the latest publications could not be provided with updated citations, which might obstruct our conclusion to some extent. It is our belief that obvious solutions to the up-to-date publications as well as non-English publications may imply a more objective evaluation.

## Conclusions

In summary, the present study overviewed the worldwide research trends with respect to sepsis and programmed cell death. As revealed in the bibliometric analysis, the United States has made great contributions in terms of the highest citation of publications, while China is superior in the number of publications. The latest research and the significant process can be traced in *Shock* and the *Journal of Immunology*. Authors including R. S. Hotchkiss, A. Ayala, C. S. Chung, and C. M. Coopersmith are identified as the most authoritative academicians in the relevant fields, who can lead the direction in this area. In the pathogenesis of sepsis, the research scope concerning immune dysregulation, oxidative stress, and inflammation, recognized as the core mechanisms of programmed cell death in sepsis, will be paid sufficient attention in the future and provide modulatory targeting strategies for the management of septic complications.

## Data availability statement

The original contributions presented in the study are included in the article/**Supplementary Material**. Further inquiries can be directed to the corresponding authors.

## Author contributions

Y-MY and Y-PT conceptualized, supervised, and revised the manuscript. J-YL and R-QY extracted all data and performed the bibliometric analysis. M-YX, Q-YZ, and P-YZ undertook and refined the searches. J-YL and R-QY drafted the paper. All authors contributed to the article and approved the submitted version.

## Funding

This work was supported by the Key Project of the National Natural Science Foundation of China (82130062, 81730057) and the Provincial Key Project of Medical Science Research of Hebei (20210013).

## Conflict of interest

The authors declare that the research was conducted in the absence of any commercial or financial relationships that could be construed as a potential conflict of interest.

## Publisher’s note

All claims expressed in this article are solely those of the authors and do not necessarily represent those of their affiliated organizations, or those of the publisher, the editors and the reviewers. Any product that may be evaluated in this article, or claim that may be made by its manufacturer, is not guaranteed or endorsed by the publisher.

## Abbreviations

WOS, Web of Science; AAY, average appearing years; PD-L1, programmed cell death ligand 1; DCs, dendritic cells; ROS, reactive oxygen species; SCI-E, Science Citation Index-Expanded; RRI, relative research interested; JCR, Journal Citation Reports; IF, impact factor; RCT, randomized clinical trial; DAMPs, damage-associated molecular patterns.
