# Supplementary figures and images for: Publication trends of research on sepsis and programmed cell death during 2002–2022: A 20-year bibliometric analysis
Source: Front Cell Infect Microbiol. 2022 Sep 23;12:999569. doi: 10.3389/fcimb.2022.999569 (PMC9537822; doi:10.3389/fcimb.2022.999569)

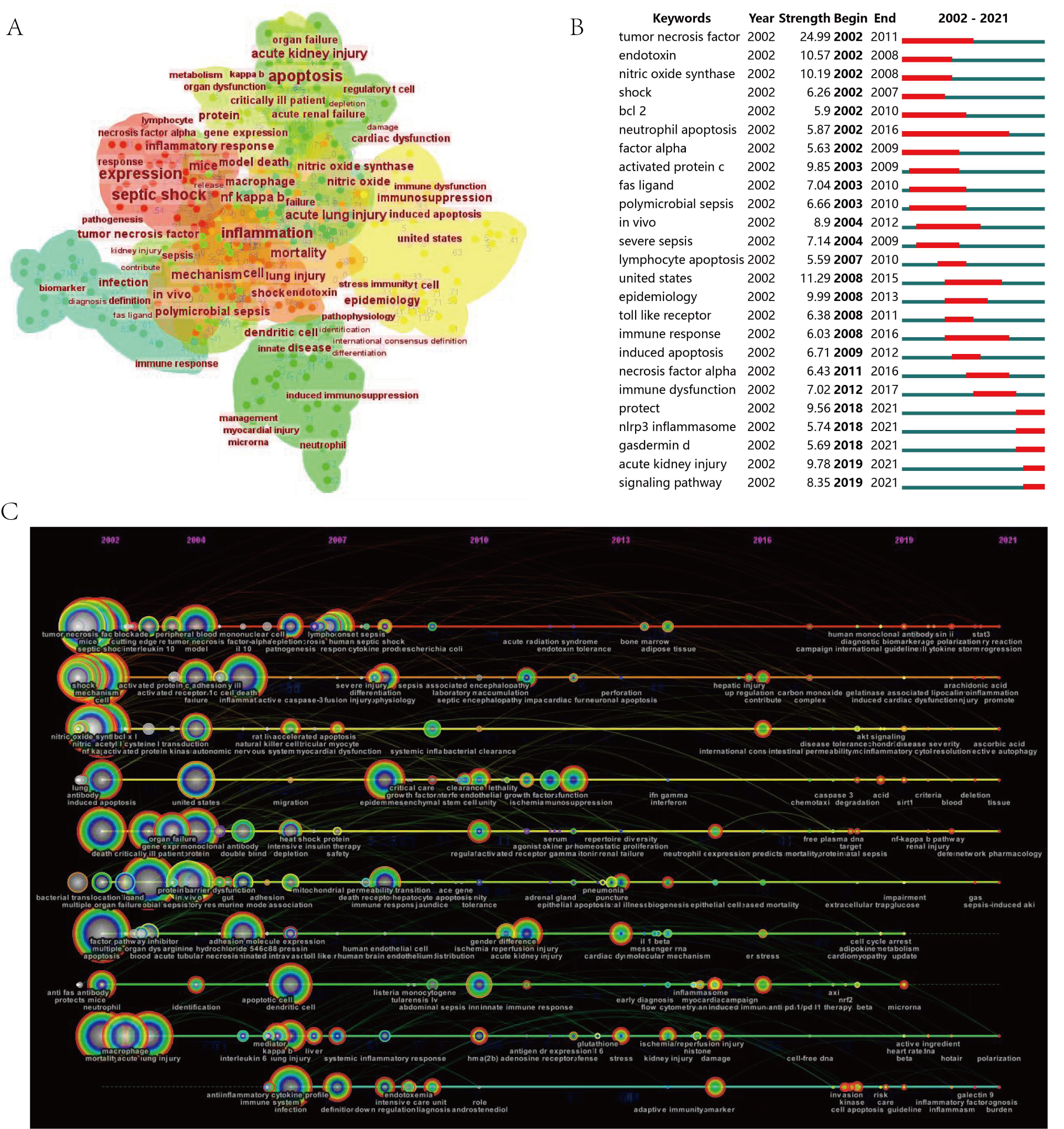

Supplement: Supplementary file 2 [file Image_1.tif]
